# Understanding the Impact of General Vaccine Attitudes on the Intent for Early COVID-19 Vaccination

**DOI:** 10.3390/vaccines11020235

**Published:** 2023-01-20

**Authors:** John Boyle, Glen Nowak, Rachel Kinder, Ronaldo Iachan, James Dayton

**Affiliations:** 1 ICF International, Rockville, MD 20850, USA; 2 Center for Health & Risk Communication, College of Journalism and Mass Communication, University of Georgia, Athens, GA 30602, USA

**Keywords:** survey research, vaccine hesitancy, attitudes and beliefs, COVID-19, nonprobability sampling

## Abstract

Despite relatively high rates of population spread, morbidity and mortality, the adoption of COVID-19 vaccines among the eligible populations was relatively slow. Some of the reasons for vaccination hesitancy and refusals have been attributed to unique aspects of this pandemic, including attitudes toward COVID-19 vaccines. However, little attention has been paid to the role of underlying vaccine beliefs in the likelihood of early vaccine adoption for COVID-19. This study provides a more comprehensive assessment of factors influencing willingness to get an early vaccination, and the relative contribution of general vaccine attitudes, compared to demographics, perceived threat and institutional trust. Monthly national surveys were conducted between June and November 2020 using a national consumer panel of U.S. adults (*n* = 6185). By late November, only 24% of respondents said they were very likely to get a Food and Drug Administration (FDA)-approved COVID-19 vaccine as soon as it became available. While COVID-19 risk perceptions, confidence and trust in key institutions and information sources, and some demographic variables, were predictive of early vaccination intent, general beliefs regarding vaccines played a significant role, even compared to demographics, perceived risk and institutional trust. This lesson from the COVID-19 experience could help inform public health communications in future epidemics.

## 1. Introduction

The first laboratory confirmed case of the 2019 Novel Coronavirus (COVID-19) in the United States was reported by the Centers for Disease Control and Prevention (CDC) on 20 January 2020 [1]. Two vaccines (Moderna and Pfizer-BioNTech) were developed for COVID-19 and found to be 95% effective in clinical trials in November 2020. The Food and Drug Administration (FDA) issued an Emergency Use Authorization for both vaccines in December 2020. At that time, the COVID-19 death toll in the United States had surpassed 300,000. In the absence of effective containment or mitigation of the virus, high vaccination coverage was widely considered critical for limiting the impact, and slowing the spread, of the virus. However, early adoption of the COVID-19 vaccines was slow in the US, despite universal eligibility for adults after the first few months, widespread availability and no cost for the vaccine. By 22 May 2021, only 57.0% of adults aged 18 or older had received at least one vaccine dose [2]. Indeed, nearly two years after the approval of these vaccines, only 80% of the US population had received at least one dose of the vaccine, and only 68% were fully vaccinated [3].

Vaccine hesitancy and refusal emerged as threats to achieving desirable levels of vaccination for COVID-19 [4,5]. Potential objections to COVID-19 vaccination include believing the benefits do not outweigh the risks or that the vaccine may be unsafe due to the accelerated development and testing timeline [6,7,8,9,10]. Inconsistent and changing government responses to COVID-19 may have eroded trust in public health, medical institutions and government and added to challenges related to convincing people to accept vaccination. Prior research involving influenza vaccine hesitancy suggests additional reasons for COVID-19 vaccine hesitancy. For example, a meta-analysis of barriers to flu vaccination found low perceived risk of serious influenza illness, perceived lack of vaccine effectiveness and benefits, and a perceived lack of incentives for getting vaccinated were the most consistent and significant vaccination barriers [11]. A national survey of U.S. born African American and White adults obtained similar results, with greater hesitancy to vaccines in general and flu vaccine specifically associated with lower rates of adult vaccination [12].

While surveys fielded in 2020 and early 2021 indicated modest overall COVID-19 vaccination intentions among the U.S. public [10,13,14], this study extended previous research about predictors of early adoption intentions for COVID-19 vaccines by examining whether general beliefs about vaccines are predictive of vaccination intentions, independently of demographics, perceptions regarding COVID-19 risks, confidence in government agencies, public health experts and information from them about the virus. This examination of the role of general beliefs about vaccines, rather than beliefs specifically about COVID-19 vaccines, in early vaccination intent is a unique aspect of this study. We conducted a review of questions entries with “vaccine” or “vaccines” in the Societal Experts Action Network (SEAN) COVID-19 Survey Archive of COVID questionnaires. The only such survey questions identified in this archive were on vaccine beliefs specific to COVID-19 or influenza, not beliefs toward vaccines in general [15]. Consequently, surveys to date have not explored core beliefs toward vaccines and whether they may play a significant role in causing hesitancy or refusals in COVID-19 vaccinations in the US. This is important in understanding whether the hesitancy to COVID-19 vaccinations is attributable to the unique aspects of the COVID pandemic and vaccine development or is more indicative of a broader public resistance to vaccinations. This, in turn, speaks to the relevancy of lessons learned from COVID-19 vaccine hesitancy for future public health programs.

A second unique contribution of this research is the assessment of the relative contribution of general vaccine beliefs, compared to perceived threat of the disease, confidence in institutions and demographics, in predicting intentions for early vaccination. If vaccine beliefs have a substantial impact on public willingness for early vaccination, this could provide primary care and other health providers critical insight in how to influence their patients’ immunization choices. This survey assessed multiple factors, including perceptions related to COVID-19 risk, as well as trust and confidence in government agencies and information. Previous research has found that the perceived threat of COVID-19 may increase immunization likelihood, while distrust of government agencies or health officials may inhibit the likelihood of such immunization [16,17,18,19]. In addition, the likelihood of COVID-19 immunization has also been related to demographic factors, including political affiliation [20,21,22].

This study thus extends previous research on COVID-19 vaccine acceptance [23,24] by using a series of repeated cross-sectional surveys that utilized early vaccination intention measures to examine (1) current beliefs toward vaccines, in general, among adults in the United States, (2) whether these general beliefs toward vaccines are predictive of COVID-19 vaccination intentions and (3) the relative contribution of general vaccine beliefs towards COVID-19 vaccine acceptance compared to risk perceptions regarding the virus, confidence in institutions to manage the virus and trust in the information about the virus, and demographic factors. We would note that one study has found a significant contribution of general vaccine beliefs in COVID-19 vaccination status in late 2021, but none have examined its role in intent for early vaccine adoption for COVID-19 [25].

## 2. Research Method

### 2.1. Participants

The data for this study are drawn from a project designed to conduct monthly national assessments of public attitudes and behaviors during the COVID-19 pandemic. In order to conduct national surveys of COVID-19 risk perceptions and behaviors, trust and confidence in government agencies and other institutions, and other pandemic associated outcomes on a monthly basis, we used a national nonprobability panel to conduct surveys in approximately one week in a cost-efficient manner [26]. The same panel, samples, questionnaire and study protocol have been used for other studies on different topics, which will include similar descriptions [25]. Respondents were drawn from the MFour mobile panel, which comprised approximately two million persons in the United States. The full panel was not designed as a representative sample, but rather as a sampling frame from which geographically and demographically representative samples can be constructed from the panel profile. Although the company says that it takes care to include traditionally underrepresented groups in its sample, we drew our monthly samples as Census balanced national samples based on the panel profile information.

The panel profile included key population characteristics of the panelist, including age, gender, race/ethnicity, education and zip code. Consequently, samples could be geographically and demographically balanced to match Census estimates. For each monthly survey, we drew a national Census-balanced (age, gender and race) sample of approximately 3000 adult panel members. An initial invitation was sent to the sample by app notification via cell phone. Non-respondents to the initial invitation were sent reminders (up to 3) via app push notification over the course of the field period. An incentive of up to $4 was offered for survey participation. Approximately 1000 interviews were completed in each wave, beginning in late March 2020. The COVID-19 vaccination intention items were added in the third month of the polling (i.e., May 2020). General vaccine belief items were added in June. The findings reported here involve the six-monthly waves conducted from June through November 2020 that included both general vaccine beliefs and COVID-19 vaccination intention items. The web-based interviews averaged 18 to 22 min in length. Monthly survey participation rates varied from 21–33%. These response rates are comparable to, if not higher than, many contemporary surveys [27,28]. Although high response rates are desirable to reduce the risk of bias in survey estimates and increase confidence in the results, research suggests that non-response bias is not necessarily associated with response rate [29]). This study was reviewed and approved by our Institutional Review Board for the protection of human subjects.

### 2.2. Survey Measures

The vaccine and vaccination measures analyzed here were part of a broader survey instrument that included items related to physical health, mental health, symptoms and treatment seeking for COVID-19, impact of the pandemic on employment and income, and demographics. Early COVID-19 immunization intention was assessed in this study with the measure: How likely are you to try to get the coronavirus (COVID-19) vaccine as soon as an FDA approved one becomes available? The four response categories were “very likely”, “somewhat likely”, “not too likely” and “not at all likely”.

We included ten general vaccine or immunization-related belief measures, which have proven useful for understanding parents’ childhood vaccination hesitancy [30]. We would note that measures such as “Some vaccines may cause learning disabilities, such as autism” and “Some vaccines have ingredients that could be harmful” do not list the range of disabilities or ingredients that might concern respondents. Rather, they represent relatively broad, but distinct, types of beliefs that the public may hold about vaccines. These vaccine beliefs were assessed using agree/disagree statements, and a scale “strongly disagree”, “somewhat disagree”, “somewhat agree” and “strongly agree”. To avoid confusion with COVID-19 related vaccine beliefs, they were introduced immediately after general health questions and questions related to seasonal flu vaccinations and before any questions related to COVID-19. The survey invitation and introduction did not mention the COVID-19 virus or the pandemic to avoid non-response bias related to the topic.

We utilized COVID-19 risk perception measures, including perceived threat to personal, family and public health, perceived risk of contracting COVID-19 and personal risk of dying from COVID-19. We also assessed confidence in the federal government, the CDC and science, as well as respondents’ trust in information from the federal government, health experts and news media (also measured as Likert scales). Items and wording for these items were drawn from or adapted from existing surveys, including the Behavioral Risk Factor Surveillance Survey (BRFSS) and published polls [14]. Most of the other dependent variables were structured as four-point ordinal variables, although risk of getting the virus and risk of dying if contracting the virus were ratio level variables ranging from 0 to 100.

Finally, information on a broad range of respondent demographics, including respondents’ political affiliation, underlying health conditions and if they believed they had been infected with COVID-19 was collected. The demographic variables included nominal (gender, race, political affiliation) and ordinal variables (age, education, and household income).

### 2.3. Statistical Analyses

We initially conducted univariate and bivariate analyses for the key dependent and independent variables. Correlations between the ten vaccine belief measures and between them and likelihood of early COVID-19 vaccine adoption were then examined. Correlation analysis was conducted using both parametric (Pearson) and non-parametric (Spearman) statistics. Since the differences in values using Pearson R and Spearman rho for all of these analyses were found to be trivial, we have presented the analyses for the more familiar parametric statistic (although non-parametric statistics are generally recommended for non-probability samples). We conducted both logistic and linear regression analyses with early vaccination intent as the dependent variable and the independent predictors from the demographic, COVID-19 related perceptions and experiences and general vaccine beliefs. We found the results of the two methods to be very comparable, with very few measures significant predictors in one model but not the other, and none of these affecting the interpretation of the findings. Although we recognize that logistic regression is the preferred statistical model for this type of analysis, we chose to present linear regression as it is more useful for a primarily non-statistician audience. The R square value as a measure of percentage of variance explained by the model is a helpful tool for many readers in evaluating the models. Similarly, the standardized beta provides an easy interpretation of the rank order of influence for the independent variables in the model (the standardized beta weight indicates the amount of change in one standard deviation in the dependent variable associated with this amount of change in the standard deviation of the independent variable). Finally, since the key independent variables and general vaccine beliefs are ordinal measures, we did not have to reduce the potential explanatory power of these variables by converting them to dichotomous measures in a logistic regression.

We conducted separate multiple linear regressions of COVID-19 vaccination intentions with independent variables from the demographic, COVID-19 specific, and general vaccine beliefs domains. A final regression of all of the independent variables on early vaccination intention was then conducted. A side-by-side comparison of linear and logistic regressions for the three domains and total model is included in Appendix B.

## 3. Results

A total of 6185 people participated in the six national surveys. Completed survey sample sizes, field periods and participation rates are given for each wave by month in Table 1. Demographics of the unweighted samples by wave are presented in Appendix A.

There were no statistically significant differences in sample demographics by wave except a marginal difference in income (*p* = 0.051). The unweighted sample had slightly fewer men (45.5% vs. 48.7%), somewhat fewer persons aged 65 and over (15.5% vs. 19.3%), somewhat more Hispanics (21.4% vs. 15.5%) and somewhat more college graduates (67.2% vs. 59.8%) than the weighted sample. The underrepresentation of those age 65+ may drive the overrepresentation of Hispanics and college graduates in the unweighted sample.

### 3.1. Early and Later Immunization Intent

In late May 2020, 34% of adults said they were “very likely” and another 29% were “somewhat likely” to try and get a FDA-approved coronavirus vaccine as soon as one becomes available. Conversely, 37% said they were “not too likely” or “not at all likely” to get an available vaccine. The percentage of respondents who were very or somewhat likely to get a vaccine as soon as available fell from 63% in May to 47% in October before rebounding modestly to 54% in November. These trends were significant at *p* < 0.001. (Table 2). These findings are consistent with findings from other published polls during this period [16,17,18]. For example, the Pew Center conducted national surveys in April/May, September and November 2020 using the question: “If a vaccine to prevent COVID-19 were available today, would you definitely get the vaccine, probably get the vaccine, probably NOT get the vaccine, or definitely NOT get the vaccine?” The proportion reporting that they would definitely/probably get the vaccine was 72% in April/May, 51% in September and 60% in November. This compares to the 63% in our survey that would be very/somewhat likely to get a COVID-19 as soon as one becomes available in May, 52.4% in September and 53.5% in November. Differences in question wording, such as the inclusion in our measure of “as soon as one becomes available” likely contributes to differences in estimates. Only 37% of adults in the Pew November survey reported that they would be comfortable in being one of the first groups of people to get the vaccine once a vaccine is approved in the US [31].

### 3.2. General Vaccine-Related Beliefs

The study found large majorities of respondents held positive beliefs about vaccines and immunizations, in general. Four of five agreed that vaccines overall were very effective (80%). Three quarters or more agreed that vaccines were important to their health (78%), being vaccinated was important to the health of the others in the community (78%) and overall vaccines are very safe (75%). A majority agreed that there was little risk in getting the disease from the vaccine (60%), and the information about vaccines from government health agencies was reliable and trustworthy (62%) (Table 3). These findings are similar to those of another study of general vaccine beliefs in August 2021 [25].

A substantial proportion of respondents did, however, hold beliefs that may produce reluctance to receive vaccines, particularly new vaccines. A majority (50%) agreed that some vaccines have ingredients that may be harmful. More than a third (34%) believed some vaccines were linked to long-term health problems and a quarter (26%) agreed that some vaccines may cause learning disabilities, such as autism. Three in ten (30%) agreed that natural infection was safer than vaccines for providing immunity.

These ten immunization-related beliefs were at least moderately correlated with one another. Among the 45 possible bivariate correlations, only one was less than 0.30, while 16 had a correlation of 0.50 or higher. One of the strongest correlations (0.684) was between beliefs that “overall vaccines are very effective” and “overall vaccines are very safe” (Table 4). Similarly, the beliefs that vaccines are important to my health and my being vaccinated is important to health of others in my community were highly correlated (0.697). The perception that some vaccines are linked to long term health problems was strongly correlated with the beliefs that some vaccines may cause learning disabilities (0.680) and some vaccines may contain ingredients that could be harmful (0.644). These findings are similar to those of the August 2021 study on vaccination outcomes [25].

Most general vaccine beliefs did not change significantly across the six months of the survey. The two exceptions were the beliefs that vaccines are important for my health, and the information about vaccines from government health agencies is reliable. In each case agreement declined by a small (i.e., 5–6 percentage points), but statistically significant, amount between June and November. This would seem to lend further credence to these general beliefs about vaccines being largely independent of dynamics of the COVID-19 pandemic.

### 3.3. Correlation of Immunization Beliefs and Intent of Early COVID-19 Immunization

We conducted bivariate correlations between general vaccine beliefs and COVID-19 vaccination intention. We found all ten general beliefs about vaccines were at least moderately correlated with the willingness to get a COVID-19 vaccine as soon as it became available, and all the correlations were statistically significant at the 0.001 level (Table 5). Although some of the vaccination belief items produced only a modest amount of “not sure” responses (i.e., 5–8%), six of them produced “not sure” responses between 13% and 28%. In a parallel analysis, the “not sure” responses were recoded to the middle of the scale between agree and disagree. This approach resulted in very little reduction in the correlations and no difference that was statistically significant. Consequently, our multivariate analyses treated “not sure” as a midpoint on the agreement/disagreement scale since this recoding minimized the loss of predictive power while including virtually all cases.

### 3.4. Predicting COVID-19 Vaccination Intention Separately with Demographics, COVID-Specific Attitudes and Experiences, and General Vaccine Beliefs

Unlike the general vaccine belief items, we found significant changes in most of the measures related to the threat of COVID-19, confidence in the federal government and the CDC and trust in information from the federal government, the CDC and news media across the six months of the survey. There were statistically significant differences across the six months in the perceived threat of COVID-19 to public and personal health, concern about family members contracting the disease and likelihood of contracting COVID-19. There were also statistically significant changes in confidence in the federal government, confidence in the CDC and trust in the information about COVID-19 from the federal government and the CDC. These changes tended to follow the average number of new cases across this six-month period. There was no statistically significant difference in confidence in scientists to act in the best interest of the public between June and November.

In the first multiple regression analysis, most of the demographic variables made a statistically significant contribution to predicting early COVID-19 vaccination intention, including gender, age, education, black race, party affiliation and residential location (rural). In line with many previous survey findings, older persons, those who were better educated and those affiliating as Democrats were more likely to intend to get an early vaccination, while those affiliating as Republicans and persons living in rural areas were less likely. However, the adjusted R square for this model was less than 0.06 (Table 6).

In the second multiple regression using COVID-19-specific attitudes to predict early vaccination intention, nine items related to perceived disease risk and institutional trust were statistically significant predictors. The perception that the coronavirus was a major threat to the respondent’s personal health was a positive predictor of early vaccination intention. Similarly, being worried about one’s family members getting the virus and the respondent’s perceived likelihood of getting the virus were both positively related to early vaccination intention. Importance of wearing a face mask to control the virus was also positively related to early vaccination intention. Confidence in the federal government and the CDC to deal with the outbreak were both positively related to early vaccination intention, as well as trust in the information from health experts and the news media about COVID-19. Confidence in science was the most powerful single predictor of early vaccination intention among these measures. Overall, the regression of these COVID-19 specific beliefs on early vaccination intention had an R square of 0.264 (Table 7).

In the third analysis, the ten general beliefs about vaccines were regressed on early COVID-19 vaccination intention. Six of the ten vaccine beliefs had a significant contribution to the prediction of early vaccination intention. Those having a positive relationship to vaccination were as follows: there is little risk of getting the disease from the vaccine, overall vaccines are very safe, vaccines are important for my health, my being vaccinated is important for the health of others in the community and information about vaccines from government health agencies is reliable and trustworthy. Only the belief that some vaccines have ingredients that could be harmful had a negative impact on early vaccination intention. Overall, the R square for this analysis was 0.258 (Table 8).

### 3.5. Combined Regression of Demographic, COVID-19 Specific and General Vaccine Beliefs

The variables from the demographic, COVID-19-specific and general vaccine belief domains were combined as independent variables in a single regression model for early COVID-19 vaccination intention. In the combined early vaccination intention model, only three demographic variables contributed significantly to the model: male gender, not black race and Democratic party affiliation. Nine measures of risk and trust associated with COVID-19 contributed significantly to the model as well, they were as follows: the virus is a threat to personal health, worried about family members’ risk, perceived personal risk of contracting COVID, perceived risk of dying from the illness, being previously sick with what they believed might be COVID-19, perceiving mask wearing as important, confidence in the federal government, trust in news media information and confidence in science. All five of the general vaccine beliefs that were significant positive predictors of early vaccination in the vaccine beliefs model remained significant when entered with demographics and COVID-19 belief variables—little risk of getting the disease from the vaccine, vaccines are very safe, vaccines are important to my health, being vaccinated is important to the health of others, the information I receive from the government about vaccines is trustworthy. Then there was the one negative predictor—some vaccines have ingredients that could be harmful—a significant negative predictor of vaccination in the full model. In sum, there were 18 variables that provided independent contributions to the prediction model for early COVID-19 vaccination intention, with an R square of 0.370 (Table 9).

## 4. Discussion

Widespread and high immunization of the American population with safe and effective vaccines is recognized as the best way to stem infectious diseases in the U.S, particularly when other public health strategies such as containment and mitigation prove ineffective. Early vaccine adoption is particularly critical to reducing deaths and serious illnesses in epidemics, once safe and effective vaccines have been approved. Achieving high vaccination rates rapidly requires understanding and addressing the key factors related to vaccine hesitancy and refusal to early vaccine adoption. Our study of the relative contribution of demographics, COVID-19 specific factors and general vaccine beliefs as predictors of early vaccination intent provides important findings and insights for future vaccination programs.

While other studies have charted vaccination intention and assessed some factors related to this intent during the COVID-19 pandemic, we believe that this is the first study to examine the contribution of general vaccine beliefs, as well as demographics and COVID specific factors, on early vaccination intentions. The focus on COVID-related factors to the exclusion of broader vaccine related beliefs limits the applicability of such findings to future epidemics and vaccination programs. The findings from this research both affirms and extends findings from previous surveys. First, as with previous surveys [16,17,18,19], the results provide important insights into associations between perceptions, demographics, and COVID-19 vaccination intentions. Consistent with other surveys [6,7,8,9,10], respondents who were men, older, white, had more formal education and indicated a Democratic affiliation were more likely to get a COVID vaccination as soon as it was available, while Republicans and those living in suburban and rural areas were somewhat less likely. This demographic profile is consistent with many reports of actual COVID-19 vaccination. However, what is notable from our study is that after controlling for general beliefs about vaccination and other COVID-19 related factors, only the male gender, non-black race, and Democratic party affiliation were significant independent predictors of early vaccination intent, and their effects were relatively small.

Second, the findings also affirm that COVID-19-related risk perceptions matter. Intentions to receive an early COVID-19 vaccination were associated with perceptions that the virus was a real health threat, including to both one’s personal health as well as one’s family. Confidence in the federal government and the CDC, along with trust in information from government health experts and news media were all positive predictors of early vaccination intent. Nine of these COVID-19 specific factors remained significant predictors of early vaccination intent, even after controlling for demographics and general health beliefs. Interestingly, the measures of confidence in science, confidence in the federal government and the importance of wearing a mask in public were each more powerful independent contributors to the likelihood of early vaccine adoption than any of the individual measures of personal threat from COVID-19 when controlling for vaccine beliefs and demographics.

Third, and most importantly, the findings related to general vaccination beliefs and early vaccine adoption, which are unique to this study, indicate that COVID-19 vaccine hesitancy for many people is significantly influenced by existing beliefs regarding vaccines. It is not just beliefs or perceptions related to COVID-19 vaccinations that influence early vaccine adoption intent. Rather, many adults held beliefs related to vaccines in general that affect their early vaccination intent for COVID-19 vaccines. Beliefs that vaccines, in general, are very safe, unlikely to transmit disease, important for personal health, important for protecting the health of others and trust in information about vaccines from government health agencies, all contributed to early vaccination intent. By contrast, concerns that some vaccines have ingredients that may be harmful negatively affect intent to get FDA-approved vaccines as soon as they are available.

The most important finding from this study is that general beliefs about vaccines are significant predictors of intent to get a COVID-19 vaccine as soon as one is available, even after controlling for demographics and COVID-19-related factors related to the threat of the disease and confidence in institutions. Indeed, five beliefs about vaccines—they are very safe, there is little likelihood of getting the disease from the vaccine, they are important for my health, they are important for the health of others in the community and the information I receive about them from the government is trustworthy—all contributed independently as positive predictors to likelihood of early vaccine adoption. One belief—some vaccines have ingredients that can be harmful—was a significant negative predictor of early vaccine adoption. Moreover, four of these general vaccine beliefs—vaccines are important for my health, my being vaccinated is important for the health of others in my community, the information I receive about vaccines from government health agencies is reliable and trustworthy, and some vaccines have ingredients that could be harmful—were more powerful predictors of early vaccine adoption than any of the measures of personal threat from COVID-19.

The role of general vaccine beliefs in intentions for early vaccination for COVID-19 has important implications for public health efforts in future disease outbreaks. Despite specific issues and concerns related to vaccine development and manufacture for COVID-19, these findings suggest that more general beliefs about vaccine safety, efficacy and benefits contribute significantly to vaccination intent. In other words, planning for vaccine education in future disease outbreaks can begin by monitoring general beliefs that affect vaccine adoption, and development and testing of communications strategies and messages to improve those beliefs.

## 5. Limitations

This study used a demographically and geographically representative non-probability consumer panel to assess immunization related beliefs and intentions during the pandemic. This approach permitted us to cost-effectively track vaccination intention, vaccine beliefs and responses to other measures over time, as well achieve samples large enough to detect differences in population segments, such as lower income households or specific racial/ethnic sub-populations, where immunization related beliefs and intentions may vary or be influenced by different factors (e.g., health information sources or access to health care resources). However, projections of estimates within statistical limits cannot be made from non-probability samples, unlike probability samples. The response rates for these surveys were substantially lower than gold standard federal surveys, but quite comparable for most other surveys. While survey estimates from non-probability surveys are not necessarily biased or unrepresentative, it is reasonable to be concerned about how low response and non-probability sample might affect survey representativeness. Nonetheless, we found the demographic characteristics of our unweighted sample were reasonably representative, even without our post-stratification weighting. Further, since we did not have to disclose topic, sponsorship or make appeals to social utility, we avoided some common response biases of probability samples. Finally, our primary goal was to examine the correlates and predictors of early vaccine intent in the population, and not point estimates of the population. Although probability samples are the gold standard for survey research, non-probability samples are increasingly accepted when factors such as a limited budget, high data collection costs or urgency make it infeasible to use a probability sample. Indeed, the CDC has conducted national non-probability web surveys to measure vaccine beliefs and intentions related to seasonal flu for a decade [32,33]. Although the non-probability sample is a limitation, we believe that this study meets the “fit-for-purpose” criteria for survey design, and there are no probability samples providing equivalent data.

## 6. Conclusions

This study demonstrated the value of including measures of general vaccine beliefs to better understand hesitancy for early adoption to COVID-19 vaccines during the pandemic in the US. Previous studies have used demographics and other COVID-19 concerns and factors to explain vaccine hesitancy and refusal during the pandemic. The inclusion of general vaccine beliefs with these other variables not only surfaced more insights related to COVID-19 hesitancy and ways to address them, but the regression analyses also demonstrated that a confluence of variables from multiple domains best predicts COVID-19 vaccination intentions. Models that only utilized demographics or COVID-related perception made more limited contributions to predicting early vaccination intentions. The most predictive model was an expansive one that better reflected the complex reality of COVID-19 vaccination decision making. The final model demonstrated that general vaccine beliefs, as distinct from beliefs more specific to COVID-19 vaccines, have a significant and substantial impact on intention for early vaccine adoption. These effects in the final model were greater than demographics and roughly equivalent to the influence of perceive threats of the COVID-19 virus and trust in government, public health and other institutions. The importance of the contribution of general vaccine beliefs to intentions for early vaccination, independent of demographics or disease specific factors, is vital to the understanding of how to design future vaccination education programs. These data suggest that success in persuading the public to adopt vaccines early in future epidemics is more likely when you know the effort must encompass general vaccination beliefs, as well as risk perceptions related to the specific disease and its public health context, and building confidence in safety, effectiveness and benefits of the specific vaccines and delivery system.

## Figures and Tables

**Table 1:** COVID-19 Monitor Surveys: Size, Dates, Response Rates, and Length.

Wave	Fielding Period	Completed Interviews	Response Rate	Interview Length
1—March	3/28/2020–4/01/2020	1000	33.3%	17:52
2—April	4/14/2020–4/23/2020	1013	28.9%	19:18
3—May	5/18/2020–5/20/2020	1002	24.1%	20:34
4—June	6/22/2020–6/29/2020	1068	23.9%	21:19
5—July	7/23/2020–7/29/2020	1014	20.6%	21:36
6—August	8/24/2020–8/31/2020	1045	20.9%	21:48
7—September	9/23/2020–10/04/2020	1060	22.0%	20:45
8—October	10/26/2020–11/01/2020	998	26.0%	22:00
9—November	11/18/2020–12/01/2020	1000	28.0%	21:49

**Table 2:** Likelihood of Getting an FDA Approved COVID-19 Vaccine as Soon as It is Available. Weighted. Missing values are excluded.

	N	Very Likely	Somewhat Likely	Not Too Likely	Not at All Likely
May	985	34.2%	29.1%	20.3%	16.3%
June	1053	31.4%	32.5%	18.5%	17.6%
July	997	30.7%	31.0%	19.8%	18.6%
August	1024	30.0%	27.1%	20.3%	22.6%
September	1040	19.7%	32.7%	20.9%	26.7%
October	992	21.1%	26.3%	25.7%	26.9%
November	988	24.0%	29.5%	23.5%	23.1%
Total	7079	27.3%	29.8%	21.2%	21.7%
Chi squared significant at 0.001 level					

**Table 3:** General beliefs toward vaccines (Weighted).

Vac. Please indicate how much you would agree or disagree with the following statements about vaccines, in general. (Excludes Prefer Not to Answer).						
	**N**	**Strongly Disagree**	**Somewhat Disagree**	**Somewhat Agree**	**Strongly Agree**	**Don’t Know/Not Sure**
VacA. Some vaccines are linked to long term health problems.	6164	19.9%	18.5%	24.2%	10.2%	27.2%
VacB. Natural infection is safer than vaccines for providing immunity.	6160	28.4%	21.4%	21.1%	8.5%	20.7%
VacC. There is little risk of getting the disease from the vaccine.	6168	9.5%	16.3%	33.9%	26.5%	13.8%
VacD. Some vaccines may cause learning disabilities, such as autism.	6158	32.1%	14.1%	17.3%	8.3%	28.1%
VacE. Some vaccines have ingredients that could be harmful.	6166	11.9%	15.5%	34.8%	15.2%	22.6%
VacG. Overall, vaccines are very safe.	6166	6.6%	11.0%	40.2%	34.4%	7.8%
VacH. Overall, vaccines are very effective.	6178	5.0%	8.1%	40.2%	39.9%	6.8%
VacI. Vaccines are important for my health	6173	7.0%	9.1%	34.4%	43.8%	5.8%
VacJ. My being vaccinated is important for the health of others in my community.	6163	6.5%	8.8%	32.4%	45.3%	7.0%
VacK. The information I receive about vaccines from government health agencies is reliable and trustworthy	6154	9.7%	15.1%	41.3%	20.8%	13.2%

**Table 4:** Correlations among General Attitudes toward Vaccines (Pairwise deletion of missing values).

Vac. Please indicate how much you would agree or disagree with the following statements about vaccines, in general.	**VacA**	**VacB**	**VacC**	**VacD**	**VacE**	**VacG**	**VacH**	**VacI**	**VacJ**	**VacK**
VacA. Some vaccines are linked to long term health problems.	1.00									
VacB. Natural infection is safer than vaccines for providing immunity.	0.502 4128	1.00								
VacC. There is little risk of getting the disease from the vaccine.	−0.348 4400	−0.321 4744	1.00							
VacD. Some vaccines may cause learning disabilities, such as autism.	0.680 4054	0.529 4107	−0.357 4370	1.00						
VacE. Some vaccines have ingredients that could be harmful.	0.644 4219	0.427 4332	−0.305 4617	0.584 4089	1.00					
VacG. Overall, vaccines are very safe.	−0.480 4541	−0.382 4981	0.533 5411	−0.487 4507	−0.422 4796	1.00				
VacH. Overall, vaccines are very effective.	−0.413 4569	−0.384 5017	0.496 5459	−0.433 4541	−0.334 4822	0.684 5843	1.00			
VacI. Vaccines are important for my health.	−0.418 4603	−0.401 5040	0.495 5471	−0.423 4559	−0.358 4876	0.650 5870	0.668 5930	1.00		
VacJ. My being vaccinated is important for the health of others in my community.	−0.385 4543	−0.401 4987	0.479 5411	−0.413 4523	−0.315 4806	0.607 5797	0.625 5865	0.697 5926	1.00	
VacK. The information I receive about vaccines from government health agencies is reliable and trustworthy	−0.373 4376	−0.271 4784	0.456 5110	−0.352 4321	−0.364 4601	0.594 5427	0.542 5456	0.549 5510	0.522 5439	1.00
All significant at 0.001										

**Table 5:** Beliefs toward vaccines and Likelihood of Adopting COVID Vaccine As Soon As Possible.

How likely are you to try and get the coronavirus (COVID-19) vaccine as soon as an FDA approved one becomes available? (Weighted) Very likely (high)						
Vac. Please indicate how much you would agree or disagree with the following statements about vaccines, in general.	Excludes Not Sure			Recodes Not Sure to Middle of the Scale		
	N	R	Sign.	N	R	Sign.
VacA. Some vaccines are linked to long term health problems.	4436	−0.292	0.001	6073	−0.253	0.001
VacB. Natural infection is safer than vaccines for providing immunity.	4838	−0.230	0.001	6068	−0.212	0.001
VacC. There is little risk of getting the disease from the vaccine.	5254	0.321	0.001	6075	0.304	0.001
VacD. Some vaccines may cause learning disabilities, such as autism.	4379	−0.271	0.001	6066	−0.231	0.001
VacE. Some vaccines have ingredients that could be harmful.	4715	−0.314	0.001	6073	−0.281	0.001
VacG. Overall, vaccines are very safe.	5610	0.409	0.001	6074	0.406	0.001
VacH. Overall, vaccines are very effective.	5682	0.372	0.001	6086	0.369	0.001
VacI. Vaccines are important for my health.	5739	0.438	0.001	6080	0.426	0.001
VacJ. My being vaccinated is important for the health of others in my community.	5653	0.418	0.001	6072	0.414	0.001
VacK. The information I receive about vaccines from government health agencies is reliable and trustworthy	5280	0.431	0.001	6063	0.409	0.001

**Table 6:** Regression of Likelihood of Vaccine Adoption (Very, Somewhat, Not Too, Not at All) by Demographics.

Early Adoption: Get Vaccine as Soon as FDA Approved Available			
Dependent Variables	N = 6018	R^2^ = 0.056	Standardized Beta (only if sign at 0.05)
Household Income			
Gender			0.101
Age			−0.043
Hispanic			
White			
Black			−0.069
Education			−0.046
Democrat			−0.178
Republican			0.033
Suburbs			
Rural			0.05

**Table 7:** Regression of Likelihood of Vaccine Adoption (Very, Somewhat, Not Too, Not at All) by Risk and Trust Factors.

	Early Adoption: Get Vaccine as Soon as FDA Approved Available		
Dependent Variables	N = 6090	R^2^ = 0.264	Standardized Beta (only if sign at 0.05)
Virus is real threat			
Threat to public health			
Threat to personal health			−0.050
Thought I had virus			
Worried about family getting sick			0.075
Likely to personally get sick			0.057
Personal risk of contracting virus (Scale)			
Personal risk of dying (Scale)			
Has underlying condition			
Important to wear face mask			−0.118
Confidence in federal govt			−0.071
Confidence in CDC			−0.052
Trust info from health experts			−0.069
Trust info from news media			−0.060
Confidence in science			0.227

**Table 8:** Regression of Likelihood of Vaccine Adoption (Very, Somewhat, Not Too, Not at All) by General Beliefs toward vaccines.

	Early Adoption: Get Vaccine as Soon as FDA Approved Available		
	**N = 5957**	R^2^ = 0.258	Standardized Beta (only if Significant at 0.05)
VacA. Some vaccine are linked to long term health problems			
VacB. Natural infection is safer than vaccines for providing immunity			
VacC. There is little risk of getting the disease from the vaccine			−0.042
VacD. Some vaccines may cause learning disabilities like autism			
VacE. Some vaccines have ingredients that could be harmful			0.113
VacG. Overall vaccines are very safe			−0.064
VacH. Overall vaccines are very effective			
VacI. Vaccines are important for my health			−0.132
VacJ. My being vaccinated is important for the health of others in my community			−0.142
VacK. The information I receive about vaccines from government health agencies is reliable and trustworthy			−0.170

**Table 9:** Regression of Likelihood of Vaccine Adoption (Very, Somewhat, Not Too, Not at All) by Demographics, COVID Risk and Trust, and General Vaccine Beliefs.

	Early Adoption: Get Vaccine as Soon as FDA Approved Available		
	N = 5064	R^2^ = 0.370	Standardized Beta (only if Significant at 0.05)
Dependent Variables			
Household Income			
Gender			0.117
Age			
Hispanic			
White			
Black			−0.045
Education			
Democrat			−0.039
Republican			
Suburbs			
Rural			
Virus is real threat			
Threat to public health			
Threat to personal health			−0.038
Thought I had virus			0.035
Worried about family getting sick			0.065
Likely to personally get sick			0.056
Personal risk of contracting virus (Scale)			
Personal risk of dying (Scale)			−0.031
Has underlying condition			
Important to wear face mask			−0.086
Confidence in federal govt			−0.083
Confidence in CDC			
Trust info from health experts			
Trust info from news media			−0.064
Confidence in science			0.120
VacA. Some vaccine are linked to long term health problems			
VacB. Natural infection is safer than vaccines for providing immunity			
VacC. There is little risk of getting the disease from the vaccine			−0.035
VacD. Some vaccines may cause learning disabilities like autism			
VacE. Some vaccines have ingredients that could be harmful			0.098
VacG. Overall vaccines are very safe			−0.041
VacH. Overall vaccines are very effective			
VacI. Vaccines are important for my health			−0.097
VacJ. My being vaccinated is important for the health of others in my community			−0.094
VacK. The information I receive about vaccines from government health agencies is reliable and trustworthy			−0.085

## Data Availability

Not applicable.

## Appendix A

**Table A1:** Sample Demographics (Unweighted) by Wave.

	NS	Total N	June	July	August	September	October	November	Total
Gender	Male	2810	42.6%	43.9%	45.9%	47.7%	46.4%	46.5%	45.5%
	Female	3365	57.4%	56.1%	54.1%	52.3%	53.6%	53.5%	54.5%
Age	18–24	782	12.5%	13.2%	12.2%	11.1%	13.4%	13.5%	12.6%
	25–34	1174	18.1%	18.5%	19.8%	19.7%	19.3%	18.4%	19.0%
	35–49	1718	27.4%	28.1%	26.7%	27.9%	27.4%	29.2%	27.8%
	50–64	1555	26.0%	23.8%	27.0%	27.0%	24.9%	21.9%	25.1%
	65+	956	15.9%	16.4%	14.4%	14.2%	14.9%	17.0%	15.5%
Education	HS Grad or less	379	6.6%	5.9%	6.0%	5.3%	6.8%	6.5%	6.2%
	Some College	1638	26.9%	25.5%	26.1%	28.0%	26.7%	26.5%	26.6%
	College grad	2158	32.5%	35.4%	34.7%	37.3%	36.8%	33.9%	35.1%
	Postgrad	1975	34.0%	33.2%	33.2%	29.4%	29.7%	33.2%	32.1%
Hispanic	Yes	1321	22.6%	21.6%	20.9%	21.3%	21.6%	20.1%	21.4%
	No	4864	77.4%	78.4%	79.1%	78.7%	78.4%	79.9%	78.6%
White race	Yes	4620	73.3%	74.6%	73.3%	77.3%	73.7%	76.0%	74.7%
	No	1565	26.7%	25.4%	26.7%	22.7%	26.3%	24.0%	25.3%
Black race	Yes	718	12.5%	10.0%	12.9%	11.1%	11.1%	12.0%	11.6%
	No	5467	87.5%	90.0%	87.1%	88.9%	88.9%	88.0%	88.4%
Household income before taxes 2019	LT $25 k	1169	18.9%	21.6%	20.0%	17.8%	19.4%	19.6%	19.6%
	25–34 k	888	15.3%	15.9%	12.8%	15.3%	14.5%	15.3%	14.9%
	35–49 k	894	14.9%	14.9%	14.0%	14.9%	15.8%	15.3%	15.0%
	50–74 k	1178	17.7%	20.2%	18.5%	20.5%	20.0%	21.3%	19.7%
	75–99 k	804	14.1%	11.6%	15.3%	12.6%	14.4%	12.7%	13.5%
	100 k+	939	17.0%	14.1%	17.8%	17.8%	14.0%	13.2%	15.7%
	Not sure	104	2.1%	1.5%	1.4%	1.0%	2.0%	2.5%	1.7%

## Appendix B

**Table A2:** Linear and Logistic Regressions of Likelihood of Vaccine Adoption (Very, Somewhat, Not Too, Not at All) by Demographics.

Early Adoption: Get Vaccine as Soon as FDA Approved Available						
Dependent Variables	Linear Regression			Logistic Regression		
	N = 6018	R^2^ = 0.056	Standardized Beta (Only If Sign at 0.05)	N = 6018	R^2^ = 0.058	Standardized Beta (Only If Sign at 0.05)
Household Income						
Gender			0.101			−0.346
Age			−0.043			0.051
Hispanic						
White						
Black			−0.069			0.418
Education			−0.046			0.101
Democrat			−0.178			0.694
Republican			0.033			−0.137
Suburbs						
Rural			0.05			−0.221

**Table A3:** Linear and Logistic Regressions of Likelihood of Vaccine Adoption (Very, Somewhat, Not Too, Not at All) by Risk and Trust Factors.

	Early Adoption: Get Vaccine as Soon as FDA Approved Available					
Dependent Variables	Linear Regression			Logistic Regression		
	N = 6090	R^2^ = 0.264	Standardized Beta (Only If Sign at 0.05)	N = 6090	R^2^ = 0.262	Standardized Beta (Only If Sign at 0.05)
Virus is real threat						
Threat to public health						
Threat to personal health			−0.050			
Thought I had virus						
Worried about family getting sick			0.075			−0.147
Likely to personally get sick			0.057			−0.142
Personal risk of contracting virus (Scale)						
Personal risk of dying (Scale)						
Has underlying condition						
Important to wear face mask			−0.118			0.455
Confidence in federal govt			−0.071			0.173
Confidence in CDC			−0.052			0.155
Trust info from health experts			−0.069			0.183
Trust info from news media			−0.060			−0.104
Confidence in science			0.227			−0.539

**Table A4:** Linear and Logistic Regression of Likelihood of Vaccine Adoption (Very, Somewhat, Not Too, Not at All) by General Beliefs toward vaccines.

	Early Adoption: Get Vaccine as Soon as FDA Approved Available					
	Linear Regression			Logistic Regression		
	N = 5957	R^2^ = 0.258	Standardized Beta (Only If Significant at 0.05)	N = 5957	R^2^ = 0.232	Standardized Beta (Only If Significant at 0.05)
VacA. Some vaccine are linked to long term health problems						
VacB. Natural infection is safer than vaccines for providing immunity						
VacC. There is little risk of getting the disease from the vaccine			−0.042			0.214
VacD. Some vaccines may cause learning disabilities like autism						
VacE. Some vaccines have ingredients that could be harmful			0.113			−0.480
VacG. Overall vaccines are very safe			−0.064			0.434
VacH. Overall vaccines are very effective						
VacI. Vaccines are important for my health			−0.132			0.440
VacJ. My being vaccinated is important for the health of others in my community			−0.142			0.583
VacK. The information I receive about vaccines from government health agencies is reliable and trustworthy			−0.170			0.696

**Table A5:** Linear and Logistic Regressions of Likelihood of Vaccine Adoption (Very, Somewhat, Not Too, Not at All) by Demographics, COVID Risk and Trust, and General Vaccine Beliefs.

	Early Adoption: Get Vaccine as Soon as FDA Approved Available					
	Linear Regression			Logistic Regression		
	N = 5064	R^2^ = 0.370	Standardized Beta (Only If Significant at 0.05)	N = 5064	R^2^ = 0.368	Standardized Beta (Only If Significant at 0.05)
Dependent Variables						
Household Income						
Gender			0.117			−0.566
Age						
Hispanic						
White						
Black			−0.045			0.413
Education						
Democrat			−0.039			0.201
Republican						
Suburbs						
Rural						
Virus is real threat						
Threat to public health						
Threat to personal health			−0.038			
Thought I had virus			0.035			
Worried about family getting sick			0.065			−0.154
Likely to personally get sick			0.056			−0.162
Personal risk of contracting virus (Scale)						
Personal risk of dying (Scale)			−0.031			
Has underlying condition						
Important to wear face mask			−0.086			0.484
Confidence in federal govt			−0.083			0.243
Confidence in CDC						
Trust info from health experts						
Trust info from news media			−0.064			0.144
Confidence in science			0.120			−0.317
VacA. Some vaccine are linked to long term health problems						
VacB. Natural infection is safer than vaccines for providing immunity						
VacC. There is little risk of getting the disease from the vaccine			−0.035			
VacD. Some vaccines may cause learning disabilities like autism						−0.205
VacE. Some vaccines have ingredients that could be harmful			0.098			−0.437
VacG. Overall vaccines are very safe			−0.041			0.398
VacH. Overall vaccines are very effective						
VacI. Vaccines are important for my health			−0.097			0.330
VacJ. My being vaccinated is important for the health of others in my community			−0.094			0.463
VacK. The information I receive about vaccines from government health agencies is reliable and trustworthy			−0.085			0.450
